# EDRIC-associated immune injury correlates with one-third of the survival detriment of elective nodal irradiation in esophageal squamous cell carcinoma

**DOI:** 10.3389/fimmu.2026.1911745

**Published:** 2026-10-26

**Authors:** Xuejiao Ren, Li Li, Xiuyuan Fan, Shuchai Zhu, Haitao Yang, Wenzhao Deng, Lihong Liu, Chun Han, Lan Wang

**Affiliations:** 1 Department of Radiation Oncology, The Fourth Hospital of Hebei Medical University, Shijiazhuang, Hebei, China; 2 Department of Epidemiology and Health Statistics, School of Public Health, and Hebei Province Key Laboratory of Environment and Human Health, Hebei Medical University, Shijiazhuang, China

**Keywords:** causal mediation, esophageal squamous cell carcinoma, estimated dose to circulating immune cells (EDRIC), host immunity, immunoprotective radiotherapy, radiation-induced lymphopenia

## Abstract

**Background and purpose:**

The survival benefit of elective nodal irradiation (ENI) over involved-field irradiation (IFI) in ESCC remains unclear, suggesting uncharacterized systemic costs. Radiation-induced lymphopenia (RIL) is increasingly recognized as an adverse prognostic factor that may undermine antitumor immunity. We investigated whether ENI increases EDRIC, a validated dosimetric biomarker of RIL, and whether EDRIC mediates ENI’s survival disadvantage via immune injury.

**Methods:**

This retrospective cohort study analyzed 279 ESCC patients treated with definitive concurrent chemoradiotherapy (2011- 2015). EDRIC was computed using a validated model incorporating mean lung, heart, and body doses and fraction number. Inverse probability weighting balanced 14 baseline covariates. Causal mediation analysis within the counterfactual framework quantified the proportion of the total effect of radiotherapy field strategy on OS mediated by EDRIC.

**Results:**

ENI significantly elevated EDRIC compared with IFI (8.17 vs. 7.42 Gy; P = 0.01). High EDRIC (>7.8 Gy) independently predicted worse OS (HR = 1.746; P<0.001), PFS, and LRFFS. The IFI- low EDRIC subgroup achieved the best 10- year OS (28.3%), significantly superior to both IFI- high EDRIC (12.0%; P = 0.004) and ENI- H group (13.5%; P = 0.024). ENI increased mortality risk by 52% (total effect Rte=1.52; P = 0.016). Causal mediation analysis revealed that EDRIC mediated 33% of this effect (indirect effect Rtnie=1.13; 95% CI: 1.02-1.29; P = 0.015).

**Conclusion:**

Elevated EDRIC was statistically associated with one-third of ENI’s excess mortality risk. Applying the exploratory 7.8 Gy median cutoff, low-EDRIC IFI was associated with superior long-term survival, suggesting a potential immunoprotective strategy that warrants further validation. These findings support optimizing radiotherapy field design to preserve host immunity in future combination regimens.

## 1 Introduction

Esophageal squamous cell carcinoma (ESCC) remains a leading cause of cancer mortality worldwide (1, 2), with 5- year survival rates below 30% despite advances in multimodality therapy (3–6). For patients with locally advanced ESCC treated with definitive concurrent chemoradiotherapy (CCRT), the optimal radiotherapy field strategy—elective nodal irradiation (ENI) versus involved-field irradiation (IFI)—has been debated for decades (7–9). While ENI offers theoretical advantages in reducing regional recurrence through prophylactic coverage of elective nodal regions, recent Phase III trials have failed to demonstrate a survival benefit over IFI, raising the possibility of unaccounted systemic costs associated with larger irradiation volumes (10).

One such systemic cost is radiation- induced lymphopenia (RIL), which has emerged as a critical determinant of oncologic outcomes across multiple solid malignancies (11–15). RIL depletes circulating T cells, B cells, and natural killer cells—key effectors of antitumor immunity—and has been associated with inferior survival and reduced response to subsequent immunotherapy. The Estimated Dose to Circulating Immune Cells (EDRIC) is a composite dosimetric parameter developed to quantify the cumulative radiation exposure to the circulating immune cell pool. It integrates mean lung dose (MLD), mean heart dose (MHD), mean body dose (MBD), and fraction number (16). In non-small cell lung cancer (NSCLC), an EDRIC >7.3 Gy has been strongly correlated with severe lymphopenia and a substantial (50%) reduction in overall survival (17). Similarly, in esophageal adenocarcinoma, an EDRIC >4 Gy was associated with higher rates of grade 4 lymphopenia (67.3% vs. 40.8%) and inferior survival (18).

We hypothesized that in ESCC, ENI, by substantially enlarging the irradiated volume, elevates EDRIC, thereby aggravating RIL and ultimately compromising survival—a relationship that may be associated with the equivocal survival benefit of ENI despite its theoretical locoregional control advantage (19–21). The growing integration of immune checkpoint inhibitors into CCRT regimens for esophageal cancer further underscores the need to understand how radiotherapy field design impacts host immunity. If ENI-induced elevation of EDRIC compromises survival by depleting circulating lymphocytes, then minimizing EDRIC through IFI or advanced radiotherapy techniques may serve as a tentative immunoprotective strategy requiring further multi-cohort verification.

This study therefore aimed to: (1) compare EDRIC between ENI and IFI in ESCC patients treated with definitive CCRT; (2) establish the relationship between EDRIC, lymphopenia, and survival outcomes; and (3) formally quantify the extent to which EDRIC mediates the association between field strategy and overall survival using causal mediation analysis.

## 2 Materials and methods

### 2.1 Study population

This retrospective cohort study was approved by the institutional review board of the Fourth Hospital of Hebei Medical University (Approval No. 2024KS209) with a waiver of informed consent. The waiver was granted because this study involved no more than minimal risk to subjects, used de-identified patient data from routine clinical practice, and it was impracticable to obtain informed consent from all eligible patients due to the retrospective nature of the data spanning a 5-year period. The study complied with the Health Insurance Portability and Accountability Act (HIPAA) and adhered to the principles of Good Clinical Practice. This study was conducted and reported in accordance with the Strengthening the Reporting of Observational Studies in Epidemiology (STROBE) statement.

We screened patients with newly diagnosed, histologically confirmed ESCC who received definitive cisplatin-based concurrent chemoradiotherapy (CCRT) at our institution between January 2011 and December 2015. Baseline demographic and clinical characteristics, including age (recorded in years), sex, Eastern Cooperative Oncology Group performance status (0–2), body weight (measured at baseline prior to treatment initiation), tumor location, clinical TNM stage (AJCC 8th edition), and cardiopulmonary comorbidity status, were systematically extracted from electronic medical records. Body mass index (BMI) was calculated as weight in kilograms divided by height in meters squared.

Key inclusion criteria were: (1) clinical stage I–IVa or IVB (with only extra-regional lymph node metastasis) according to the American Joint Committee on Cancer (AJCC) 8th edition TNM staging system; (2) treatment with intensity-modulated radiation therapy (IMRT) to a total dose of 50.4–66 Gy using conventional fractionation (1.8–2.0 Gy per fraction); (3) Eastern Cooperative Oncology Group (ECOG) performance status of 0–2; (4) absence of major organ dysfunction, pre-existing immune system diseases, or prior malignancies. Patients with non-squamous histology, multiple primary cancers, treatment interruptions lasting ≥1 week, or missing essential clinical or dosimetric data were excluded. Of the initial screened population (n=391), 112 patients were excluded: 25 had non-squamous histology, 15 had multiple primary cancers, 9 had treatment interruptions lasting ≥1 week, and 63 had missing dosimetric or essential clinical data. No patients were lost to follow-up within the first 5 years; after 5 years, annual follow-up was completed for 267 patients (96.0%), with 12 patients lost to follow-up due to relocation or change of contact information (attrition rate: 4.0%). Based on these criteria, 279 eligible patients were included and categorized into IFI (n=141) and ENI (n=138) groups based on radiotherapy field strategy.

Sex was recorded for all patients based on medical records. The study population included 192 male (68.8%) and 87 female (31.2%) patients (see **Table 1**). Sex was included as a covariate in the propensity score model and multivariable analyses to adjust for potential sex-specific differences in treatment outcomes.

**Table 1:** Baseline characteristics of 279 ESCC patients treated with IFI or ENI before and after IPTW.

	Before IPTW					After IPTW				
	Overall (n=279)	IFI (n=141) %	ENI (n=138) %	P-value	SMD	Overall (n=279)	IFI (n=141) %	ENI (n=138) %	P-value	SMD
Sex				0.904	0.013				0.548	-0.039
Male	192 (68.8)	98 (69.5)	94 (68.1)			190.7 (68.8)	96.5 (67.8)	94.2 (71.7)		
Female	87 (31.2)	43 (30.5)	44 (31.9)			82.9 (31.2)	45.8 (32.2)	37.1 (28.3)		
Age (years)	66 ± 8.8	67 ± 9.2	64 ± 7.9	0.002	-0.416	64 ± 9.2	65.8 ± 10.6	62.1 ± 8.5	0.953	0.008
ECOG PS				0.001	-0.133				0.913	-0.006
1	246 (88.2)	115 (81.6)	131 (94.9)			243.1 (88.8)	126.0 (88.5)	117.1 (89.1)		
2	33 (11.8)	26 (18.4)	7 (5.1)			30.7 (11.2)	16.4 (11.5)	14.3 (10.9)		
Cardiopulmonary Comorbidity				0.002	-0.189				0.936	-0.005
Yes	139 (49.8)	57 (40.4)	82 (59.4)			140.8 (51.4)	72.8 (51.1)	68.0 (51.7)		
No	140 (50.2)	84 (59.6)	56 (40.6)			133 (48.6)	69.6 (48.9)	63.4 (48.3)		
Tumor Location				<0.001	-0.252				0.868	-0.011
Cervical/Upper Thoracic	110 (39.4)	38 (27.0)	72 (52.2)			106.6 (38.9)	54.6 (38.4)	52.0 (39.6)		
Middle/Lower Thoracic	169 (60.6)	103 (73.0)	66 (47.8)			167.1 (61.1)	87.7 (61.6)	79.4 (60.4)		
Endoscopic Lesion Length (cm)	7.3 ± 2.7	7.6 ± 2.9	6.9 ± 2.4	0.189	-0.215	6.9 ± 4.8	7.3 ± 4.6	6.6 ± 4.6	0.548	-0.091
GTV Volume (cm³)	42.7 ± 31.1	45.0 ± 36.8	40.4 ± 23.9	0.554	-0.150	40.8 ± 45.6	42.7 ± 37.5	38.8 ± 32.3	0.716	-0.048
cT Stage				0.014					0.862	
T2	87 (31.2)	36 (25.5)	51 (37.0)		0.114	87.5 (32.0)	43.1 (30.2)	44.4 (33.8)		0.035
T3	117 (41.9)	71 (50.4)	46 (33.3)		-0.170	111.5 (40.7)	58.9 (41.3)	52.6 (40.1)		-0.012
T4	75 (26.9)	34 (24.1)	41 (29.7)		0.056	74.8 (26.9)	40.4 (28.4)	34.4 (26.2)		-0.022
cN Stage				0.022					0.989	
N0	61 (21.9)	26 (18.4)	35 (25.4)		0.069	62.3 (22.8)	32.9 (23.1)	29.4 (22.4)		-0.007
N1	127 (45.5)	59 (41.8)	68 (49.3)		0.074	119.6 (43.7)	62.6 (44.0)	57.0 (43.4)		-0.005
N2	66 (23.7)	37 (26.2)	29 (21.0)		-0.052	67.5 (24.7)	33.7 (23.6)	33.8 (25.7)		0.020
N3	25 (9.0)	19 (13.5)	6 (4.3)		-0.091	24.2 (8.8)	13.1 (9.2)	11.1 (8.5)		-0.007
cM Stage				0.036	-0.110				0.766	0.019
M0	219 (78.5)	103 (73.0)	116 (84.1)			212.7 (78.5)	111.9 (78.6)	100.8 (76.7)		
M1	60 (21.5)	38 (27.0)	22 (15.9)			61 (21.5)	30.4 (21.4)	30.6 (23.3)		
cTNM Stage				0.05					0.776	
I	2 (0.7)	2 (1.4)	0 (0.0)		-0.014	1 (0.37)	1.0 (0.7)	0.0 (0.0)		-0.007
II	67 (24.0)	38 (17.7)	42 (30.4)		0.127	62.6 (22.9)	31.9 (22.4)	30.7 (23.3)		0.009
III	79 (28.3)	30 (30.5)	36 (26.1)		-0.044	73.5 (26.9)	39.4 (27.6)	34.1 (25.9)		-0.017
IV	131 (47.0)	71 (50.4)	60 (43.5)		-0.068	136.7 (49.9)	70.1 (49.3)	66.6 (50.7)		0.014
Chemotherapy Cycles				<0.001	0.254				0.769	0.021
1 cycle	135 (48.4)	86 (61.0)	49 (35.5)			127.2 (46.5)	67.6 (47.5)	59.6 (45.4)		
2 cycle	144 (51.6)	55 (39.0)	89 (64.5)			146.5 (53.5)	74.7 (52.5)	71.8 (54.6)		
Radiation Dose				<0.001	0.239				0.550	0.039
<60Gy	50 (17.9)	42 (29.7)	8 (5.7)			42.1 (15.4)	24.6 (17.3)	17.5 (13.3)		
≥60Gy	229 (82.1)	99 (70.2)	130 (94.3)			231.7 (84.6)	117.8 (82.7)	113.9 (86.7)		
Baseline Lymphocyte Count (×10^9^/L)	1.7 ± 0.5	1.7 ± 0.6	1.6 ± 0.5	0.095	0.109	1.6 ± 1.3	1.8 ± 1.4	1.5 ± 0.9	0.469	0.048

Data are presented as n (%) or mean ± SD. IPTW, inverse probability of treatment weighting; SMD, standardized mean difference; ECOG PS, Eastern Cooperative Oncology Group performance status; GTV, gross tumor volume; cT/cN/cM, clinical tumor/node/metastasis stage according to the AJCC 8th edition; cTNM, clinical TNM stage according to the AJCC 8th edition. SMD < 0.1 indicates good balance after IPTW. Baseline lymphocyte count is expressed as ×10^9^/L.

### 2.2 Radiation treatment planning

Target volumes were delineated consistently according to institutional protocols. The Gross Tumor Volume (GTV) included the primary tumor (GTV-t) and involved lymph nodes (GTV-n). GTV-t was defined based on imaging criteria, including esophageal wall thickness >0.5 cm or a non-air-filled wall diameter >1.0 cm, as assessed by barium esophagography, endoscopy, and correlative computed tomography (CT) or positron emission tomography (PET)-CT imaging. GTV-n included lymph nodes with a short axis diameter ≥1.0 cm on CT, or nodes in specific locations (e.g., para-esophageal, tracheoesophageal groove) with a long axis ≥0.5 cm, or smaller nodes exhibiting suspicious morphology (e.g., round shape, necrosis, or high avidity on PET-CT). The Clinical Target Volume (CTV) encompassed CTV-t (GTV-t with 2–3 cm longitudinal and 0.5–0.8 cm radial margins) and CTV-n. For IFI, CTV-n was generated by expanding the GTV-n isotropically by 0.3–0.5 cm, confined strictly to the involved nodal region. For ENI, elective coverage of specific, at-risk lymphatic drainage areas was mandated based on the primary tumor location (cervical, upper, middle, or lower thoracic), encompassing relevant nodal stations as per established guidelines (10). The Planning Target Volume (PTV) was generated by uniformly expanding the respective CTV by 0.5 cm in all dimensions to account for setup uncertainties and organ motion.

Organs at risk (OARs) were contoured and constrained according to standard guidelines: lung V5 ≤55–60%, V20 ≤25–30%, V30 ≤18–20%; spinal cord maximum dose <45 Gy; heart mean dose ≤30 Gy. All treatment plans were generated and delivered using IMRT techniques. Chemotherapy consisted of FP (cisplatin 25 mg/m² days 1–3, 5-fluorouracil 450–500 mg/m² days 1–5) or TP (paclitaxel 135 mg/m² day 1, cisplatin 25 mg/m² days 2–4) administered concurrently during weeks 1 and 5 of radiotherapy.

### 2.3 Outcome assessment and EDRIC calculation

The primary survival endpoint was overall survival (OS), defined as the time from the initiation of CCRT to death from any cause. Secondary survival endpoints included progression-free survival (PFS), local-regional failure-free survival (LRFFS), and local control (LC), assessed at 3, 5, and 10 years. Adverse events were graded according to the Common Terminology Criteria for Adverse Events (CTCAE) version 4.0, and specific radiation toxicities were graded per the Radiation Therapy Oncology Group (RTOG) criteria. Estimated Dose to Circulating Immune Cells (EDRIC) was a validated dosimetric biomarker of radiation exposure to the circulating immune cell pool, which was calculated for each patient using the previously validated formula (17):


$$\text{EDRIC}=0.12\times \text{MLD}+0.08\times \text{MHD}+\left[0.45+0.35\times 0.85\times {\left(\text{n}/45\right)}^{\wedge }\left(1/2\right)\right]\times \text{MBD}$$

where MLD is mean lung dose, MHD is mean heart dose, MBD is mean body dose, and *n* is the number of fractions. Importantly, this EDRIC formula was originally derived and validated in non-small cell lung cancer and has not been formally calibrated for ESCC.

### 2.4 Statistical analysis

All statistical analyses were performed using R software (version 4.1.3; R Foundation for Statistical Computing, Vienna, Austria; RRID: SCR_001905). This was a retrospective cohort study, and thus patients were not randomly assigned to the IFI or ENI groups; treatment decisions were made based on institutional protocols and physician discretion based on tumor location and nodal involvement. To mitigate selection bias and confounding by indication, we employed inverse probability of treatment weighting (IPTW). Propensity scores were estimated using a logistic regression model that included 14 pre-specified baseline covariates. Covariate balance between the IFI and ENI groups before and after IPTW application was assessed using standardized mean differences (SMD), with an SMD <0.1 indicating adequate balance.

Outcome assessors and statisticians were blinded to the radiotherapy field assignment during the analysis phase. Clinical data were abstracted from electronic medical records by trained research assistants who were not involved in treatment planning and were blinded to the study hypothesis. The primary endpoint (overall survival) was objectively defined as death from any cause, which is not subject to assessment bias.

Survival analyses were performed using the Kaplan-Meier method, and between-group differences were compared using the log-rank test. Univariable and multivariable Cox proportional hazards regression models were used to estimate hazard ratios (HRs) and 95% confidence intervals (CIs) for the associations of EDRIC, field strategy, and other clinical factors with survival endpoints. The proportional hazards assumption for Cox regression models was tested using Schoenfeld residuals (global test P = 0.31), with no significant violation detected. Notably, the IPTW model separately included cT, cN and cM as three independent covariates to finely balance baseline features (14 total covariates). For multivariable Cox regression, we merged the three separate stage variables into one composite TNM stage, additionally adjusted for three post-treatment toxic adverse events, and incorporated two core study predictors (radiation field strategy, EDRIC grouping), resulting in a total of 17 adjusting factors.

Causal mediation analysis was conducted within the counterfactual framework using the cmest function from the CMAverse package in R (version 0.1.0). This analysis decomposes the total effect (TE) of the radiotherapy field strategy (ENI vs. IFI) on OS into a natural direct effect (NDE; effect not through EDRIC) and a natural indirect effect (NIE; effect mediated through EDRIC). The models were adjusted for the same baseline covariates used in IPTW and included an exposure-mediator interaction term. Effect estimates (expressed as risk ratios, RR) were derived via the imputation approach, and 95% CIs were computed based on 20, 000 non-parametric bootstrap samples. The proportion mediated (pm) was calculated as ln(NIE)/ln(TE). Note that this log-ratio-based proportion-mediated estimator for the risk-ratio scale should be interpreted with caution; it is not identical to the risk difference-based proportion mediated. All statistical tests were two-sided, and P values <0.05 were considered statistically significant. Exact p-values are reported in the results section; for p<0.001, we report as p<0.001.

This was a retrospective cohort study using a consecutive sample of eligible patients treated between 2011 and 2015; therefore, sample size was determined by the availability of eligible patients rather than *a priori* power calculation. A *post-hoc* power analysis indicated that the observed sample sizes (n=141 in the IFI group and n=138 in the ENI group) provided approximately 80% power to detect a hazard ratio of 1.5 for overall survival at a two-sided alpha level of 0.05, assuming a baseline 5-year survival rate of 25%. However, *post hoc* power calculations have well-known limitations and should be interpreted cautiously. Patient follow-up was conducted regularly every 1 to 6 months via outpatient clinic visits for the first 5 years and annually thereafter, supplemented by telephone calls or home visits when necessary. Patients lost to follow-up after 5 years were censored at the time of last contact.

### 2.5 Data and code availability

#### 2.5.1 Data availability

The datasets generated and/or analyzed during the current study are not publicly available due to institutional data protection policies and patient privacy regulations, but are available from the corresponding author upon reasonable request. The de-identified patient data supporting the findings of this study are available from the corresponding author, subject to institutional review board approval and a signed data access agreement.

#### 2.5.2 Code availability

The R code used for the inverse probability of treatment weighting, causal mediation analysis, and survival analyses in this study is available from the corresponding author upon reasonable request.

## 3 Results

### 3.1 Patient characteristics and survival outcomes

Baseline characteristics of the 279 eligible ESCC patients before and after IPTW adjustment are summarized in **Table 1**. Before IPTW, several covariates were imbalanced between the IFI and ENI groups, including age, ECOG performance status, cardiopulmonary comorbidity, tumor location, cT stage, cN stage, cM stage, chemotherapy cycles, and radiation dose (all P < 0.05). After IPTW, all 14 baseline covariates were well-balanced between the two groups, with all SMDs < 0.1, indicating successful mitigation of selection bias (**Table 1**).

For the entire cohort, the 3-, 5-, and 10-year OS rates were 37.4%, 27.6%, and 20.0%, respectively. The corresponding rates for PFS were 29.3%, 21.0%, and 15.9%; for LRFFS were 30.0%, 21.9%, and 17.2%; and for LC were 45.9%, 38.4%, and 32.3%. Following IPTW adjustment, we observed no statistically significant differences in OS, PFS, LRFFS, or LC between the IFI and ENI groups when analyzed based on the radiotherapy field strategy (**Figures 1A–D**).

**Figure 1:**
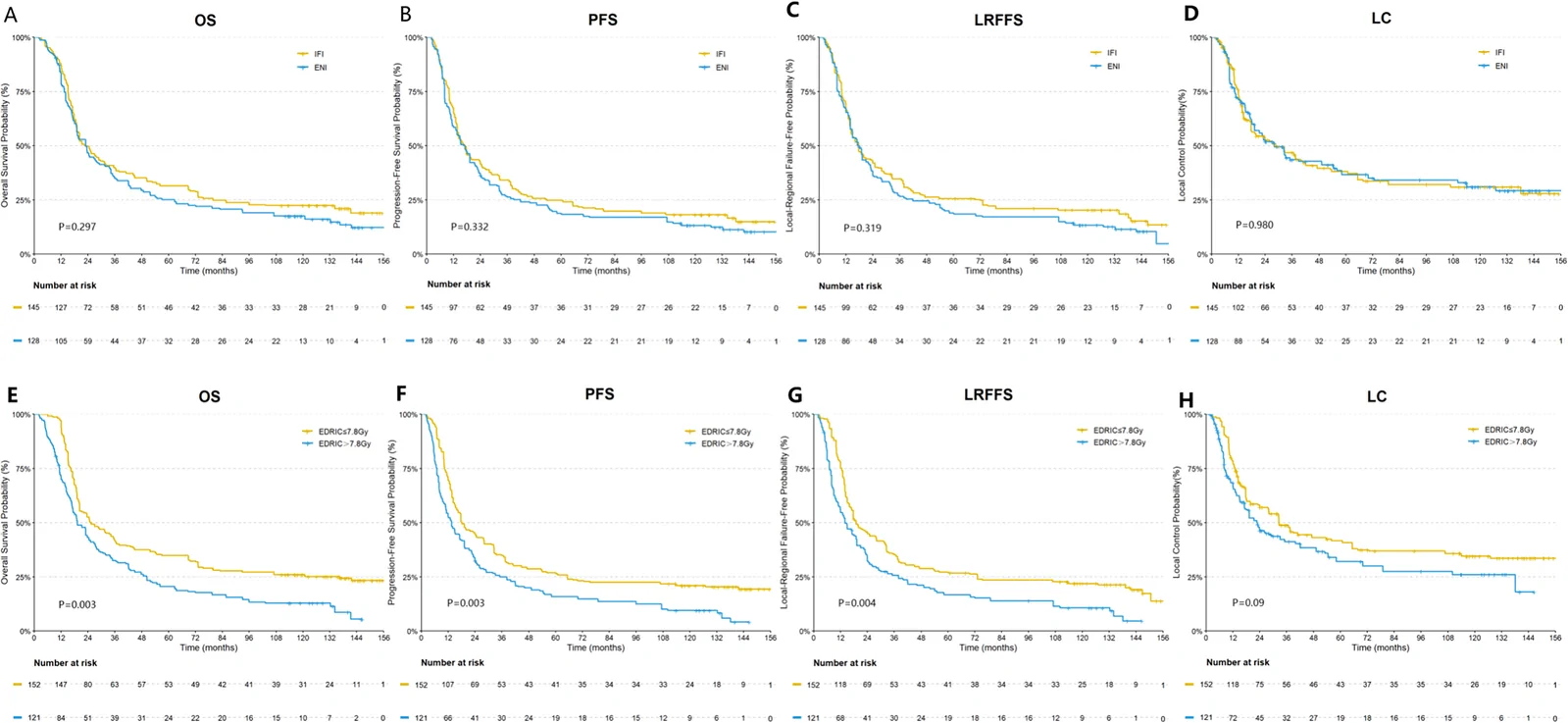
Kaplan-Meier curves for survival endpoints stratified by radiotherapy field strategy and EDRIC level. OS **(A)**, PFS **(B)**, LRFFS **(C)** and LC **(D)** for patients receiving involved-field irradiation (IFI) versus elective nodal irradiation (ENI) after IPTW adjustment. OS **(E)**, PFS **(F)**, LRFFS **(G)** and LC **(H)** for patients stratified by low (≤7.8 Gy) versus high (>7.8 Gy) EDRIC. Log-rank P values are presented.

### 3.2 EDRIC distribution and correlations

The mean EDRIC for the entire cohort was 7.79 ± 1.95 Gy (range: 3.00–12.18 Gy). The baseline absolute lymphocyte count averaged 1.67 ± 0.53 ×10^9^/L (range: 0.58–3.85 ×10^9^/L), which declined sharply during treatment to a nadir (LN) of 0.34 ± 0.16 ×10^9^/L (range: 0.03–1.07 ×10^9^/L). The mean PTV volume was 422.34 ± 198.97 cm³ (range: 27.85–1367.84 cm³). The ENI group had a significantly larger mean PTV volume compared to the IFI group (547.05 ± 174.96 cm³ vs. 300.27 ± 135.35 cm³, P = 0.001). The mean EDRIC was also significantly higher in the ENI group (8.17 ± 1.92 Gy vs. 7.42 ± 1.91 Gy, P = 0.01). The LN showed a non-significant trend toward lower values in the ENI group (0.32 ± 0.14 ×10^9^/L vs. 0.36 ± 0.17 ×10^9^/L, P = 0.08). Spearman correlation analysis revealed a significant positive correlation between PTV volume and EDRIC across all patients (r = 0.387, P < 0.001; **Figure 2A**), and this correlation remained significant within both the IFI (**Figure 2B**) and ENI (**Figure 2C**) subgroups. Furthermore, we observed a significant inverse correlation between EDRIC and LN for the entire cohort (r = −0.355, P < 0.001; **Figure 2D**), and this relationship was consistently significant within both the IFI (**Figure 2E**) and ENI (**Figure 2F**) subgroups.

**Figure 2:**
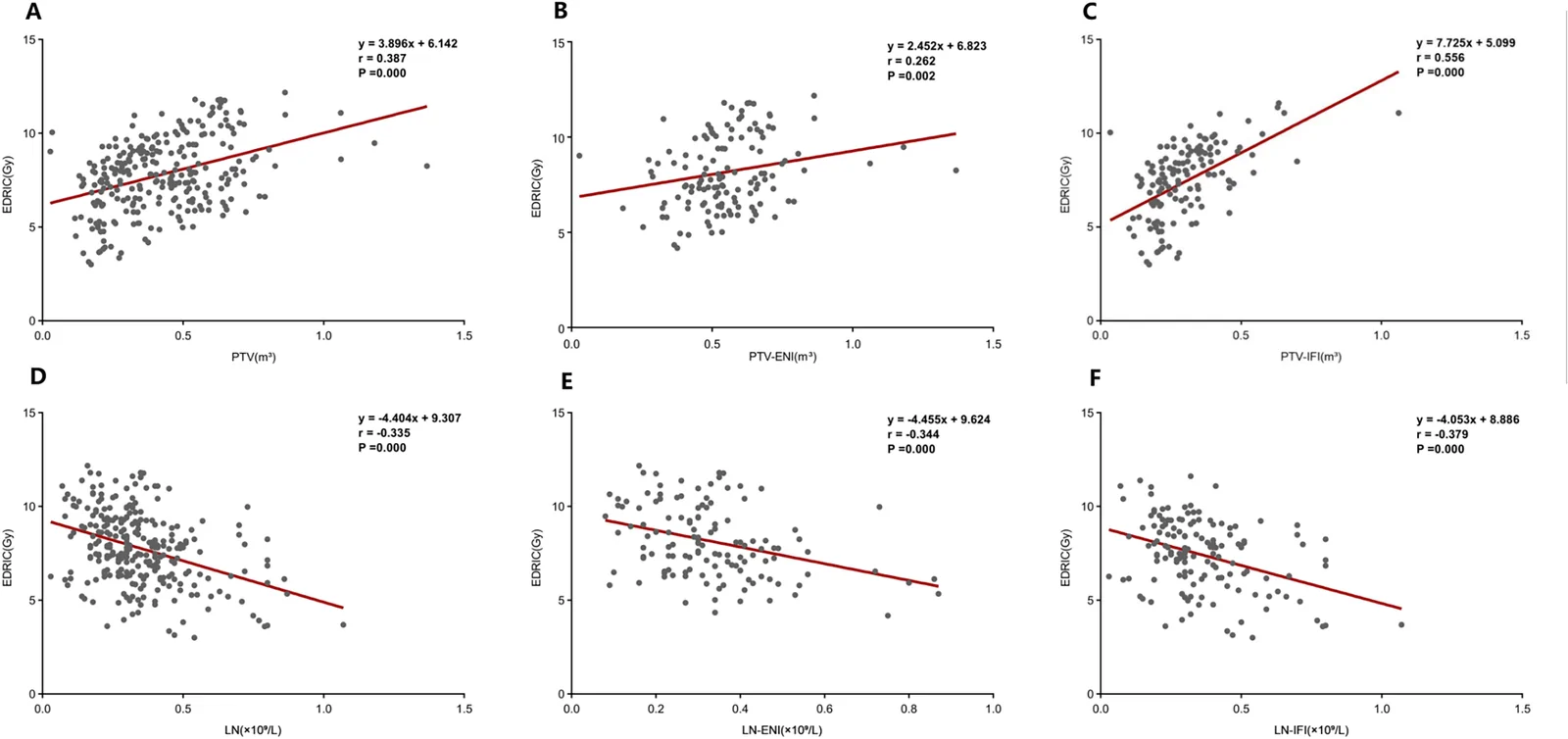
Correlations between Planning Target Volume (PTV), Estimated Dose to Circulating Immune Cells (EDRIC), and Lymphocyte Nadir (LN). **(A–C)** Scatter plots demonstrating the positive correlation between PTV volume and EDRIC for **(A)** all patients, **(B)** ENI group, and **(C)** IFI group. **(D–F)** Scatter plots demonstrating the inverse correlation between EDRIC and LN for **(D)** all patients, **(E)** ENI group, and **(F)** IFI group. Correlation coefficients (r) and P-values are from Spearman rank correlation analysis.

### 3.3 Survival analysis stratified by EDRIC and field strategy

Using the cohort median EDRIC (7.8 Gy) as an exploratory cutoff, we stratified patients into low (EDRIC-L, ≤7.8 Gy) and high (EDRIC-H, >7.8 Gy) EDRIC groups for hypothesis-generating analyses. The EDRIC-L group exhibited significantly superior OS, PFS, and LRFFS (P = 0.003, 0.003, 0.004; **Figures 1E–G**), with 10-year rates of 26.0% vs. 12.9% (OS), 21.0% vs. 9.4% (PFS), and 22.0% vs. 10.6% (LRFFS) for EDRIC-L vs. EDRIC-H, respectively. We found no significant difference in LC between the two groups (P = 0.09; **Figure 1H**).

To further elucidate the interplay between field strategy and EDRIC, we cross-stratified patients into four groups: IFI-L, IFI-H, ENI-L, and ENI-H. Significant differences among these four groups were observed for OS (P = 0.004), PFS (P = 0.008), and LRFFS (P = 0.01) (**Figures 3A–C**), but not for LC (P = 0.200; **Figure 3D**). Detailed 3-, 5-, and 10-year survival rates for each subgroup are provided in **Table 2**. Pairwise comparisons demonstrated that the IFI-L group had the most favorable outcomes, with the 10-year OS (28.3%) superior to both the IFI-H group (12.0%, P = 0.004) and ENI-H group (13.5%, P = 0.023). Similarly, IFI-L had significantly better PFS and LRFFS compared to IFI-H (P = 0.009, P = 0.023) and ENI-H (P = 0.021, P = 0.020). We observed no significant survival differences between ENI-L and ENI-H, or between IFI-H and ENI-H.

**Figure 3:**
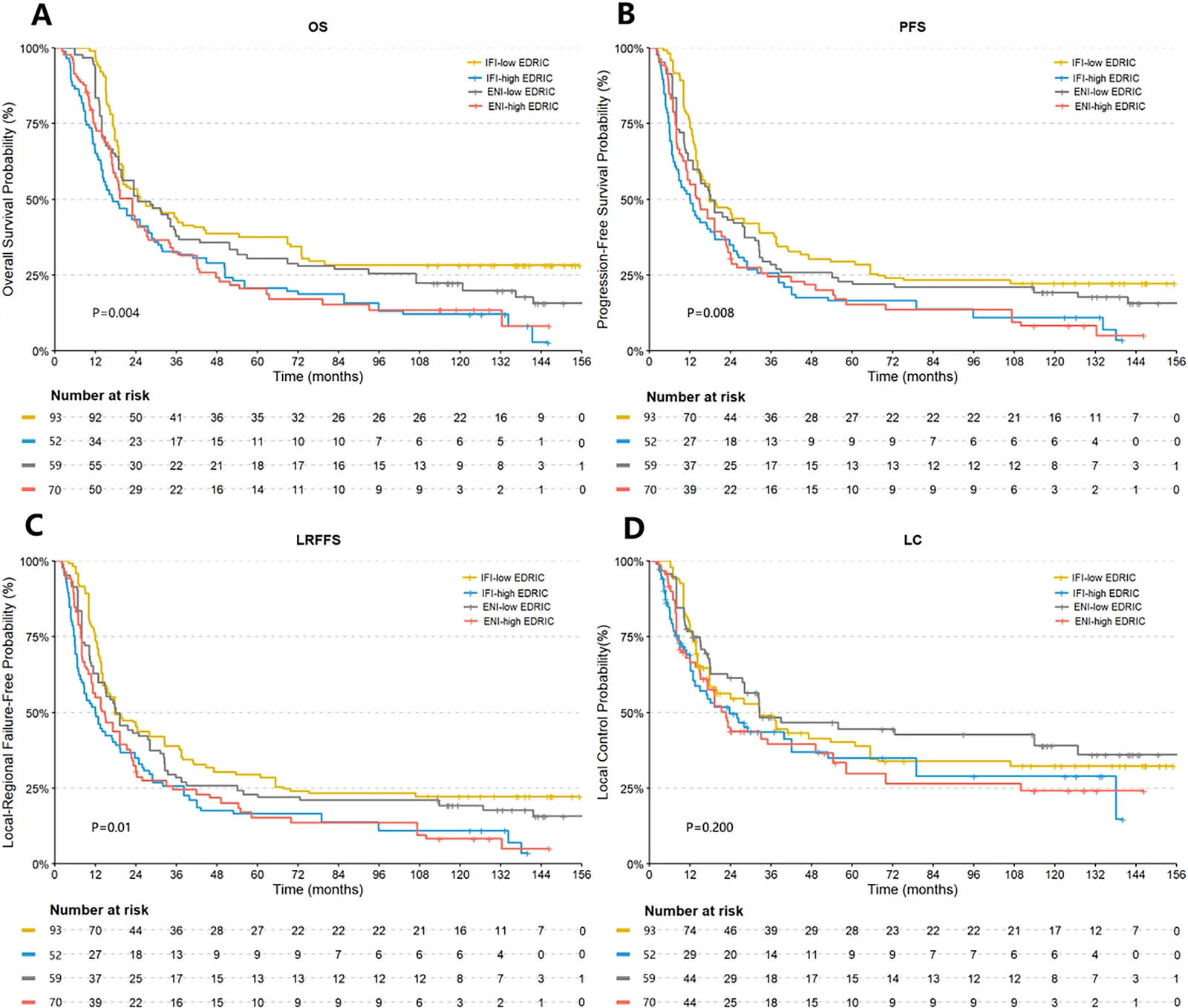
Kaplan-Meier survival curves for the four subgroups defined by combined radiotherapy field strategy and EDRIC Level. OS **(A)**, PFS **(B)**, LRFFS **(C)** and LC **(D)** for the four patient subgroups: IFI-L (involved-field irradiation with low EDRIC), IFI-H (involved-field irradiation with high EDRIC), ENI-L (elective nodal irradiation with low EDRIC), and ENI-H (elective nodal irradiation with high EDRIC). Log-rank P values are presented.

**Table 2:** Comparison of 3-, 5-, and 10-year survival outcomes across subgroups defined by field strategy and EDRIC level.

Groups	OS (%)				PFS (%)				LRFFS (%)				LC (%)			
	3-y	5-y	10-y	P value	3-y	5-y	10-y	P value	3-y	5-y	10-y	P value	3-y	5-y	10-y	P value
IFI-L	44.0	37.6	28.3	0.004	38.8	29.4	22.2	0.008	38.8	29.4	23.4	0.01	48.9	40.1	32.3	0.200
IFI-H	32.7	20.6	12.0		25.7	16.5	11.0		27.5	18.8	14.6		43.6	34.9	28.9	
ENI-L	30.4	28.8	22.3		28.5	22.9	19.2		29.1	23.4	19.7		48.3	44.5	39.0	
ENI-H	32.3	20.5	13.5		24.5	15.2	8.2		24.5	15.2	8.2		40.0	29.8	24.1	

OS, overall survival; PFS, progression-free survival; LRFFS, local-regional failure-free survival; LC, local control; IFI-L, involved-field irradiation with low EDRIC; IFI-H, involved-field irradiation with high EDRIC; ENI-L, elective nodal irradiation with low EDRIC; ENI-H, elective nodal irradiation with high EDRIC. Log-rank P values are presented.

Multivariate Cox regression, adjusting for 17 clinical factors, identified high EDRIC (>7.8 Gy) as an independent adverse prognostic factor for OS (HR = 1.746, 95% CI: 1.327–2.295, P < 0.001). The development of grade 4 lymphopenia during treatment was also independently associated with worse OS (HR = 1.415, 95% CI: 1.002–1.999, P = 0.049). Furthermore, high EDRIC was independently associated with inferior PFS (HR = 1.713, 95% CI: 1.315–2.231, P < 0.001), LRFFS (HR = 1.577, 95% CI: 1.155–2.152, P = 0.004), and LC (HR = 1.749, 95% CI: 1.274–2.066, P = 0.001).

### 3.4 Causal mediation analysis

The results of the causal mediation analysis are summarized in **Table 3**. In contrast to the marginal IPTW analysis above (Section 3.1), this mediation analysis estimates conditional effects by adjusting for the mediator (EDRIC), the exposure-mediator interaction term, and all baseline covariates. This framework decomposes the total effect of ENI versus IFI into an indirect effect mediated through EDRIC and a direct effect independent of EDRIC, thereby revealing effect structures that may be masked in undivided marginal survival comparisons. Specifically, the IPTW-adjusted Kaplan-Meier analysis estimates a marginal population-averaged effect based on crude group-level survival without conditioning on the mediator, whereas the mediation model is conditional and estimates a conditional total effect rather than a marginal effect. Thus, the non-significant marginal survival difference and the significant conditional total effect are not contradictory, because they address different estimands. These two analyses are complementary: the former assesses whether an overall group-level survival difference is detectable, while the latter quantifies how the total effect is partitioned into EDRIC-mediated and EDRIC-independent pathways. The analysis confirmed a significant total effect of ENI (vs. IFI) on increased mortality risk (Total Effect Rte = 1.52, 95% CI: 1.08–2.16, P = 0.016). Crucially, this detrimental effect was partially mediated by EDRIC. The indirect effect, representing the path through which ENI increases mortality risk by elevating EDRIC, was statistically significant (Total Natural Indirect Effect Rtnie = 1.13, 95% CI: 1.02–1.29, P = 0.015). The proportion of the total effect mediated by EDRIC was 33% (pm = 0.33, 95% CI: 0.049–1.16, P = 0.030). This suggests EDRIC as a potential mediator of the survival difference between ENI and IFI. A direct effect of the field strategy independent of EDRIC was also observed but was marginally non-significant (Pure Natural Direct Effect Rpnde = 1.35, 95% CI: 0.96–1.87, P = 0.083; Total Natural Direct Effect Rtnde = 1.37, 95% CI: 0.94–1.98, P = 0.099), suggesting the involvement of additional, unmeasured mechanisms beyond EDRIC contributing to the survival detriment of ENI.

**Table 3:** Causal mediation analysis of radiotherapy field strategy on overall survival via EDRIC.

Effect measure	Relative risk/effect ratio	95% CI	P value
Controlled Direct Effect (Rcde)	1.29	0.82- 1.98	0.276
Pure Natural Direct Effect (Rpnde)	1.35	0.96- 1.87	0.083
Total Natural Direct Effect (Rtnde)	1.37	0.94- 1.98	0.099
Pure Natural Indirect Effect (Rpnie)	1.11	1.00- 1.26	0.040
Total Natural Indirect Effect (Rtnie)	1.13	1.02- 1.29	0.015
Total Effect (Rte)	1.52	1.08- 2.16	0.016
Proportion Mediated (pm)	0.33	0.049- 1.16	0.030

Rcde, controlled direct effect; Rpnde, pure natural direct effect; Rtnde, total natural direct effect; Rpnie, pure natural indirect effect; Rtnie, total natural indirect effect; Rte, total effect; pm, proportion mediated. Effects are expressed as relative risks (RR) with 95% confidence intervals(CIs). Models were adjusted for baseline covariates and included an exposure–mediator interaction term. Bootstrap-derived CIs are based on 20, 000 replicates.

## 4 Discussion

This study supports the hypothesis that EDRIC may serve as a mediator linking elective nodal irradiation to survival detriment in ESCC. Using causal mediation analysis within a counterfactual framework, we demonstrate that ENI increases mortality risk by 52%, with approximately one-third (33%) of this effect attributable to elevated EDRIC—a dosimetric biomarker of radiation exposure to circulating immune cells. Patients receiving IFI with EDRIC ≤7.8 Gy achieved favorable 10-year overall survival of 28.3%. This internally generated exploratory median threshold needs further validation via external cohorts and prospective clinical investigations.

Mechanistically, ENI substantially expands the irradiated volume (mean PTV volume: 547.05 cm³ vs. 300.27 cm³ for IFI), significantly increases the estimated immune cell dose (EDRIC: 8.17 Gy vs. 7.42 Gy). This elevated EDRIC, in turn, exacerbates treatment-induced lymphopenia and ultimately compromises overall survival. This finding may provide immunologic insight for the long-standing clinical controversy surrounding the optimal radiotherapy field strategy in ESCC, wherein ENI has failed to consistently demonstrate a survival benefit over IFI in modern trials despite its broader target coverage (7–10, 22–25).

### 4.1 Relation to the growing field of radio- immunology

Our results align with the growing recognition that radiation’s effect on host immunity is a critical determinant of therapeutic success. RIL is not merely a benign laboratory finding but a clinically impactful immune-depleting event that can offset the locoregional benefits of more extensive radiotherapy (16–18, 26, 27). The EDRIC threshold of 7.8 Gy identified in our definitive CCRT cohort for ESCC is notably higher than the 4 Gy threshold reported for esophageal adenocarcinoma treated with concurrent chemoradiotherapy (18) or the 7.3 Gy threshold for stage III NSCLC treated definitively (17). Other studies have reported thresholds of 10.3 Gy for grade 4 lymphopenia in ESCC (27) and 6.86 Gy in the neoadjuvant setting (28). This discrepancy likely reflects intrinsic differences in treatment paradigms, including typically higher radiotherapy doses (≥60 Gy in 82.1% of our cohort) used in definitive settings for ESCC, as well as the distinct anatomical distribution of tumors (primarily middle/lower thoracic in our cohort) influencing dose to circulating blood through major cardiopulmonary vessels. This underscores the cancer type-, stage-, and treatment modality-specific nature of immune radiosensitivity and the need for specific EDRIC optimization (28, 29). The consistent dose-response relationship observed in our study—where 10-year OS plummeted from 26.0% to 12.9% when EDRIC exceeded 7.8 Gy—powerfully reinforces the clinical validity and prognostic utility of this metric. It also complements the recent findings by Cai et al. (27), who described a dose-gradient effect, with EDRIC ≥10.3 Gy being linked to a 55.2% rate of grade 4 lymphopenia in a similar patient population.

### 4.2 Beyond lymphopenia: implications for locoregional control and immune surveillance

A key mechanistic insight from our analysis is the identification of high EDRIC as an independent risk factor not only for OS and PFS but also for local-regional control (LRFFS: HR = 1.577). This finding challenges the conventional radiotherapy paradigm that predominantly emphasizes maximizing tumor-target dose for local control, suggesting instead that systemic immune injury inflicted by large-volume irradiation can indirectly foster local and regional recurrence. A recent comprehensive review delineates the intricate multicellular interplay driving radiotherapy resistance within the tumor microenvironment, with hypoxia, stromal remodeling and immune escape as core cascading mediators, emphasizing that radiation-induced TME reprogramming is not merely characterized by lymphocyte depletion, but relies on complex cross-talk among diverse stromal and immune populations to fuel tumor recurrence and therapeutic failure (30). Consistent with this view, we hypothesize that the underlying immunosuppressive cascade triggered by high EDRIC may be multifactorial, potentially involving the depletion of circulating effector lymphocytes, impaired regeneration of lymphoid organs, thymic atrophy (31), remodeling of the tumor immune microenvironment with Treg/MDSC/PD-L1 changes (32), and perturbation of the gut microbiota–immune axis (33). However, none of these mechanisms were directly measured in this study; they remain hypotheses for future serial immune-monitoring studies.

### 4.3 Clinical translation: immunoprotective radiotherapy

From a clinical and practical standpoint, our results strongly advocate for a paradigm shift towards “immune-protective radiotherapy” in the management of ESCC. The superior long-term survival observed in the IFI-L group (10-year OS: 28.3%) underscores the substantial therapeutic advantage achievable by minimizing radiation dose to the circulating immune system. This can be achieved by adopting IFI whenever clinically feasible, particularly in cases without extensive, high-risk nodal basins. For cases when ENI is necessary (e.g., multi-station lymph node involvement, high risk of subclinical disease), or even within IFI plans, rigorous attention should be paid to constraining doses to organs that significantly contribute to EDRIC, primarily the lungs and heart. Implementing strict dose-volume constraints (e.g., lung V20 <25%, mean heart dose <10-15 Gy, heart V40 <30%) during IMRT planning is crucial to keep low EDRIC, without compromising tumor coverage (e.g., PTV coverage ≥95%). For patients with high-risk features necessitating ENI, advanced radiotherapy techniques should be actively explored to mitigate immune cell irradiation. These include partial lymphatic sparing approaches, which strategically omit elective irradiation of certain low-risk nodal stations; proton therapy, which exploits its physical Bragg peak characteristic to significantly reduce integral dose and exposure to circulating blood (34–36); and potentially ultra-high dose rate FLASH radiotherapy (37).

### 4.4 Implications for immunotherapy combinations

The implications of our findings extend to the burgeoning field of immunotherapy combination strategies. As ongoing trials like KEYNOTE-975 (38) evaluate the integration of PD-1 inhibitors with definitive CCRT for esophageal cancer, preserving a functional immune system by minimizing EDRIC-induced lymphopenia may be critical for optimizing synergistic efficacy. Severe lymphopenia during chemoradiotherapy can potentially blunt the subsequent immune response to checkpoint blockade (28). The recent report by Ma et al. (39)—demonstrating that an EDRIC >6.26 Gy increases mortality risk by 20.7% in patients with NSCLC receiving postoperative radiotherapy—further underscores the universal importance of preserving the host immune system across different cancer types and treatment modalities. Beyond lymphopenia, emerging evidence indicates that immune cell metabolic states can drive cancer treatment resistance, with metabolic reprogramming of tumor-infiltrating lymphocytes and macrophages influencing both the efficacy of immunotherapy and the response to combined chemoimmunotherapy regimens. This immunometabolic viewpoint further reinforces the clinical value of EDRIC-minimizing radiotherapy to maintain intact anti-tumor immune function (40). Recent translational research utilizing multi-marker immune phenotyping has further demonstrated that dynamic shifts in functional CD8+ resident/bystander T cells and conventional CD4+ T subsets strongly correlate with pathological response after neoadjuvant chemoimmunotherapy. This reinforces that merely tracking bulk lymphocyte depletion fails to characterize the functional status of intratumoral immune competence; serial profiling of tumor-infiltrating T-cell subpopulations is required to evaluate precisely how immunotherapy-based regimens modulate anti-tumor immunity (41). Our study provides a quantitative estimate of the extent to which survival loss from large-field radiotherapy is statistically associated with immune depletion, offering a rationale for prospectively testing EDRIC- guided radiation planning in the immunotherapy era.

## 5 Limitations

Several limitations warrant consideration. First, despite IPTW adjustment for 14 covariates, residual confounding inherent to retrospective studies cannot be entirely excluded. Prospective validation of the EDRIC threshold in a randomized controlled trial is needed. Second, the EDRIC formula, while validated in lung cancer (16, 42), has not been specifically calibrated for ESCC. Developing an ESCC- specific algorithm incorporating tumor location- specific blood flow patterns would enhance precision. Third, IFI with EDRIC ≤7.8 Gy is an exploratory immunoprotective strategy identified in our single-center cohort. This internally derived cutoff requires external validation, time-dependent ROC analysis, and continuous EDRIC sensitivity testing before clinical application. Fourth, while we propose low EDRIC as an immunoprotective target, prospective studies correlating EDRIC with post-treatment immune cell subset analysis are needed to validate the underlying immunobiology. Fifth, the proposed immunological mechanisms were not directly measured and should be considered hypothesis-generating. Notably, all mediation proportions reflect statistical associations rather than definitive biological causation. Future prospective, multicenter randomized trials are therefore necessary to establish whether the observed mediation effects represent true causal pathways.

## 6 Conclusions

This single-center observational study provides associative evidence that elevated EDRIC correlates with approximately one-third of the excess mortality risk associated with ENI in ESCC. IFI with EDRIC ≤ 7.8 Gy represents an exploratory immunoprotective radiotherapy approach derived from our internal cohort; this median cutoff requires external validation and prospective trials before any clinical adoption in chemoimmunoradiotherapy regimens. Our findings suggest that radiotherapy field design can be optimized as an immunomodulatory strategy to preserve host antitumor immunity while maintaining locoregional tumor control.

## Data Availability

The datasets generated and/or analyzed during the current study are not publicly available due to institutional data protection policies and patient privacy regulations, but are available from the corresponding author upon reasonable request. The de-identified patient data supporting the findings of this study are available from the corresponding author, subject to institutional review board approval and a signed data access agreement.
